# Postnatal Depression and Its Associated Factors in Women From Different Cultures

**Published:** 2011

**Authors:** Fatemeh Abdollahi, Munn-Sann Lye, Azhar Md Zain, Sazlina Shariff Ghazali, Mehran Zarghami

**Affiliations:** 1 Department of Public Health, College of Health, Mazandaran University of Medical Sciences, Sari, Iran,; 2 Department of Community of Health, Faculty of Medicine and Health Sciences, University Putra Malaysia, Serdang, Malaysia; 3 Department Psychiatry, Faculty of Medicine and Health Sciences, University Putra Malaysia, Serdang, Malaysia; 4 Department of Family Medicine, Faculty of Medicine and Health Sciences, University Putra Malaysia, Serdang, Malaysia; 5 Department of Psychiatry and Psychiatry and Behavioral Sciences Research Centre, Mazandaran University of Medical Sciences, Sari, Iran

**Keywords:** Cultures, Postpartum depression, Risk factors

## Abstract

**Objective:** Postpartum depression (PPD) is a common health problem which affects women in the postpartum period. This is a brief note on its associated factors in women from different cultures.

**Methods:** A literature review was performed in MEDLINE and Pubmed from 1991 to 2008 and Magiran from 1991 to 2009. Additional articles and book chapters were referenced from these sources.

**Results:** The prevalence of postpartum depression has been reported to be from 0.5% to 60% globally, and from 3.5% to 63.3% in Asian countries, in which Malaysia and Pakistan had respectively the lowest and highest rates. One of the factors contributing to PPD in Asian societies can be that women may not have the empowerment to reject traditional rituals that are imposed on them by their caregivers. Unsatisfactory pre-existing relationships between the mothers and their caregivers resulting in mothers experiencing difficulties during their confinement period may be another factor. Thirdly, some features of these traditional rituals may be the cause of tension, stress and emotional distress. Emotional conflicts caused by insistence on practice of traditional rituals during the postpartum period may lead to mental breakdown.

**Conclusion:** Health care professionals should be aware that the phenomenon in Asian cultures is as prevalent as European cultures. Moreover, further research needs to be conducted on the global prevalence of the experiences of childbearing women with depressive symptoms.

## Introduction

Over the past four decades, many studies have emphasized the importance of mood disorders after childbirth (1). Postpartum depression (PPD) is more frequent in women than thought otherwise (2). Unfortunately, little attention has been paid to this condition in terms of identification, diagnosis, and treatment (3). Mothers at risk are seldom identified during pregnancy or at the time of delivery (4). This occurs especially in developing countries, where psychological issues are mostly ignored (5). It should be noted that up to 80% of cases do not seek medical attention and thus, are not diagnosed by the respective specialists (6).

Cultural dimensions play a significant role in the perception and experience of motherhood in variety of cultures. The diversity of prevalence of postpartum depression across the cultures may assist researchers in understanding whether this disorder is primarily brought on by psychological or biological factors (7).

*Prevalence of PPD*

Recently, there appears to be a growing international recognition of postnatal depression as a significant public health concern (8). It has been noted that postnatal depression, particularly in western countries, affect 10-15% of postpartum women (9, 10), but many researches and epidemiological studies have recognized the occurrence of an increasingly high incidence of PPD in diverse cultures in different parts of the world (11). In a review by Halbreich and Karkun (2006) of 140 past and related studies from 40 countries, the reported frequency of PPD was from 0.5% to over 60% (5). These studies revealed that in some societies, namely Singapore, Malta, Denmark and Malaysia, the prevalence of PPD was quite low (0.5-9%), while it was very common in other countries such as Guyana, Costa Rica, Italy, Chile, South Africa, Korea and Taiwan (34-57.0%). This variation has been reported to be about 20-30% in the north (12,13) and more than 40% in the West (14) and East (15) of Iran.

Halbreich and Karkun (2006) reported that the frequency of PPD of 10-15% is unlikely to be an accurate global indication of this problematic issue (5). Their opinion mainly relied on two observations; firstly, it was based on a wide variation of cited prevalence which ranges from 0% to more than 60%. It is worthwhile to note that this inter-country variation does not completely cover all the within-country cross-cultural and diversified socio-economic situations, rendering the estimate hard to interpret. Secondly, most researches have used the Edinburgh Postnatal Depression Scale (EPDS) to measure PPD. The EPDS screening is focused on postpartum mood disorder which does not take into consideration anxiety, irritability and other symptoms that have been shown to be recurrent among the women, particularly during reproductive-related periods. Hence, EPDS may not be able to detect the considerable wide range of pre- and postpartum symptoms and disorders (5).

Another issue in the application of EPDS might be the variability of cut-off scores across countries, which in these examples ranged from 9 to 13. EPDS developers, who suggested a range of 9-10 to 13-14 for different populations, also recommended culturally sensitive cut-off points. Thus, EPDS cut-off scores are different in different cultures along with the sensitivity and specificity of the instruments that have been developed (5,16).

Errors in the estimation of the prevalence of PPD can be attributed to the inadequate sensitivity and specificity of the instrument (5). Gaynes et al. found in their review that sensitivities and specificities of EPDS at all evaluated thresholds [12, 13, 14, 15] were 1.0 and from 0.79 (at EPDS >12) to 0.96 (at EPDS >15) for major depressive disorder. Sensitivity was much poorer (0.57 to 0.71) and specificity remained fairly high (0.72 to 0.95) for minor depressive disorder (17).

*Culture and PPD*

Since culture provides a significant context for all human experiences and is comprised of several shared ideas, perspectives, cognitive styles, and standards for emotional and behavioral responses, it can truly affect the way any individual comes to experience depression and consequently the way s/he asks for support whether physical or emotional. In this sense, one cannot deny the role of culture as an important factor in pregnancy and postpartum adjustment (18).

Dankner et al. (2000) were of the opinion that cultural elements such as definition of roles, community support and rituals could explain the existing discrepancies in PPD (19). In some traditional environments, the range of postpartum depression is less than other settings. This phenomenon highlights the significance of cultural patterns which strengthen maternal role transition and as a result, may reduce the physical and psychological tensions of the new mother. Dankner et al. (2000) who conducted a research in Jewish Jerusalem women in Israel observed a decreasing tendency in EPDS mean scores in cross-secular, traditional, religious and orthodox families (19).

Asia as the largest and most populous continent in the world containing four billion people is categorized into six separate regions, namely Central Asia (including countries such as Uzbekistan, Kazakhstan), East Asia (including China, Hong Kong, Japan), South-east Asia (including Thailand, Malaysia, Singapore, East Timor, Vietnam), and finally West Asia (including Iran, Turkey, Israel, Unites Arab Emi-rates) (20). These regions encompass a multitude of languages, socioeconomic settings; cultural history, religious rituals and varying status of mental health that inevitably affect the mother in the postpartum period (20). Some cultures practice traditional rituals and helpful methods which are believed by their members to have an effective outcome in supporting and protecting women from the possible symptoms of depression (21).

Klainin and Gordon Arthur (2009) showed that the frequency of PPD in Asian countries ranged from 3.5% to 63.3% (22). It is noteworthy that Malaysia and Pakistan had respectively the lowest and highest frequency of occurrence. It has been shown that traditional postpartum rituals cannot be considered as a preventive factor nor are they always of psychological benefit for the new mothers.

Factors affecting PPD in Asian countries can be classified into five main groups:

• Physical/biological factors, namely medical condition of the mother and premenstrual symptoms, body mass index (BMI) below normal, and food consumption with high levels of riboflavin (vitamin B2), and high dietary glycemic index.

• Psychological factors, namely symptoms  of depression during the pregnancy, antenatal anxiety, past psychiatric history.

• Obstetric/ pediatric factors, namely complications during pregnancy, experience of abortion, previous loss of a baby, unplanned pregnancy.

• Sociodemographic factors, namely financial problems, experiencing hunger in the past month, being a homemaker.

• Cultural factors, namely different habits of taking bath, washing one’s hair, going out of the house, food habits and even being blown by the wind (22).

The first three groups mentioned above were consistent with the results of the previous meta-analytic researches which were mainly based on Western populations (9, 10, 22-24).

Important factors of PPD in those studies included prenatal depression, history of depression, psychiatric disorders during pregnancy, childcare anxiety, stressful life incidences, low self- respect, low social support, low marriage satisfaction, unintended pregnancy, difficult infant temperament and low socioeconomic status (9, 10, 23, 24).

The first three groups mentioned above were consistent with the results of the previous meta-analytic researches which were mainly based on Western populations (9, 10, 22-24). Important factors of PPD in those studies included prenatal depression, history of depression, psychiatric disorders during pregnancy, childcare anxiety, stressful life incidences, low self-respect, low social support, low marriage satisfaction, unintended pregnancy, difficult infant temperament and low socioeconomic status (9, 10, 23, 24).

Anthropologists declare that the high frequency of PPD in Western societies is a justification for the claim that PPD is a “culture-bound syndrome”. Western culture promotes individualistic responsibility, and the social isolation caused by urbanization and economic restrictions predispose mothers to higher rates of depression (25). Other researchers proposed that the very absence of birth rituals and the general decrease in domestic support (for instance, fragmentation of families and the increasing number of the close relatives who have occupations away from home) will consequently result in the increasing rate of the depression (18). 

In countries which have been influenced by the Western values, once the baby is born, most of the attention and the care of family and those of the health care providers would usually be cast upon the baby itself and the mother would tend to be neglected. In quite the opposite pole, other cultures put much more emphasis on the new mother and her physical and emotional needs, while certain parenthood rites supply special protection for her (7). Furthermore, inadequacy of a suitable role and lack of support for the new mothers may create a milieu for development of depression (26).

At this juncture, it is worthwhile mentioning the results of an international study by Affonso et al. (2000) which indicates lower mean scores of depressive symptomatology in Western European and higher mean levels in the Asian participants (27). The researchers point out that the improvement and prevailing programs for PPD besides the education and treatment programs in European societies may very effectively decrease the possibility of depressive symptomatology. Results demonstrated the prevalence rate of PPD in Sweden is lowest (between 13% and 15.2%), USA comes in between (between 37% and 29.5%), while Taiwan has the highest rates (between 73.7% and 60.8%) at 4 and 6 weeks postpartum respectively (27).

*Recent changes of PPD prevalence*

Scholars affirm that there is a close relation between the decreasing risk of PPD and the availability of education and treatment programs provided for new mothers in European and Australian societies. In contrast to these results, higher mean levels of depressive symptomatology and absence of such programs in Asian and South American societies indicate that PPD in those countries is not treated seriously as a health concern (8).

Previous and recent studies have shown that increasing prevalence of PPD is not limited to western and industrialized countries. In the past three decades, societies in developing countries have experienced rapid demographic and socioeconomic transformation, and the elimination of the traditional structure, which may be an influencing factor in increasing the prevalence of postnatal disorders in women in these countries, although more research needs to be conducted in this area (28).

Another factor for the occurrence of PPD in developing societies may be the conflicting recommendations from the part of the family members and the professionals in health care. There may be sometimes quite different opinions and demands from the family regarding the care of baby and mother. Often it causes considerable stress when mothers follow the traditional structures while at the same time health care professionals with their contradictory beliefs impose their own advice that seldom seem to be reasonable for the mothers (29).

Another element that may possibly have an impact on the prevalence of PPD is the lack of postnatal supportive equipment and services and cross-cultural variation in return visits following delivery in many countries that will eventually affect the rates of PPD in different societies (5). In some cultures, mothers choose to depend on traditional treatment before resorting to medical care. Therefore, their labor may be more difficult and painful and this may increase the risk of PPD (30).

It should not be overlooked that maternal depression is sometimes postponed to later stages of the postpartum period. Accordingly, depression in the second and third months of postpartum period may be due to the mother’s response to the withdrawal of postnatal support and the sometimes bitter acceptance of the realities of motherhood (5, 31).

In some cultures, women experiencing depressive symptoms under-utilize healthcare services because of the stigma these mothers undergo. These mothers sometimes go into denial, while some of them assume that these symptoms are a common phenomenon of childbirth, or are momentary problems that will eventually be ameliorated (32).

*Cultural elements in traditional societies*

A variety of postpartum rites such as the prescribed confinement periods which normally range from 30 to 40 days, controlled activities and diets, and practical/emotional support from family members, mother, and mother-in-law, traditional birth attendant, or female relatives are practiced in Asian communities (29).

These cultural rituals have their own pros and cons and two opposite outcomes. This kind of support can be effective in providing physical comfort. On the other hand, it can act as a significant source of mental conflict and emotional disturbance. A number of researches indicate that postpartum rituals in Japan, Vietnam, Malaysia, Hong Kong, and Singapore could not by any means provide considerable psychological advantage for the new mothers (22).

The results of a systematic review of the role of traditional practice in the reduction of PPD by Wong and Fisher (2009) showed that confinement could not be assumed to be available to, welcomed by or effective in all the Chinese participants (33).

Klainin and Gordon Arthur (2009) proposed that there are four probable reasons for such unpredicted results. Firstly, the women may have decided to practice the traditional rituals by their own choice; rather it was imposed on them by their caregivers (e.g., mother-in-laws). Secondly, due to unsatisfactory pre-existing relationships between the mothers and their caregivers, mothers may experience interpersonal difficulties during their confinement period. Thirdly, some features of these traditional rituals, mainly the restricted practices, may cause tension, stress and emotional disturbance. Finally, inevitable challenges including cultural issues, for instance gender preferences and the longing for male offspring and baby’s health condition and temperament, new mothers’ physical/ psychological make-up, and economical and financial status of the family in the postpartum situation and conflict with traditional rituals may cause mothers undue psychological distress. Many studies have reported that women, who have had higher levels of education and have experienced other forms of cultures, are often resistant to their own traditional cultures (22).

## Conclusion

It is noteworthy that the variation in the range of postnatal depression prevalence reported by researchers is considerable since it depends on factors such as instruments used, the methods in which mothers were defined as ‘’depressed’’, the quality of the translation of study materials from English to the source language, the study design, sampling methods, differences in symptom definition and expression, and the duration after delivery when the depression is evaluated. In addition, it may be unclear if depression is a new occurrence or carried over from depression that occurred during pregnancy (5, 21).

Finally, it should be emphasized that health care professionals should be aware that the phenomenon in Asian cultures is as prevalent as in European cultures. Although numerous studies have been conducted on PPD, further research is needed to be performed on the experiences of childbearing women with depressive symptomatology globally.

## Authors' Contributions

FA conceived and designed the evaluation and helped to draft the manuscript. M-S L, A Md Z, SSG advised on the study design and planning of the study, re-evaluated the data, advised on the analysis and revised the manuscript. MZ advised on the study design and planning of the study, collected some of the data, re-evaluated the data, and revised the manuscript. All authors read and approved the final manuscript.

## Notes

**Declaration of Interest:** None.

## Notes

*In some traditional environments, the range of postpartum depression is less* *than other settings. This phenomenon highlights* *the significance of cultural patterns which* *strengthen maternal role transition and as a result,* *may reduce the physical and psychological tensions of the new mother.*

*In countries which have been influenced by the western values, once the baby is born, most of the attention and the care of family and those of the health care providers would usually be cast upon the baby itself and the mother would tend to be neglected.*

*In quite the opposite pole, other cultures put much more emphasis on the new mother and her physical and emotional needs, while certain parenthood rites supply special protection for her.*

*Cultural rituals have their own pros and cons and two opposite outcomes. This kind of support can be effective in providing physical comfort. On the other hand, it can act as a significant source of mental conflict and emotional disturbance.*
